# Development of a novel model for intraarticular adhesion in rat knee joint

**DOI:** 10.1371/journal.pone.0292000

**Published:** 2023-09-21

**Authors:** Ryo Nakahara, Akira Ito, Akihiro Nakahata, Momoko Nagai-Tanima, Hideki Kawai, Kisara Uchiyama, Kohei Nishitani, Tianshu Wang, Tomoki Aoyama, Hiroshi Kuroki

**Affiliations:** 1 Department of Motor Function Analysis, Human Health Sciences, Graduate School of Medicine, Kyoto University, Kyoto, Japan; 2 Japan Society for the Promotion of Science, Tokyo, Japan; 3 Department of Orthopaedic Surgery, Graduate School of Medicine, Kyoto University, Kyoto, Japan; University of Life Sciences in Lublin, POLAND

## Abstract

In this study, a novel rat model of knee joint adhesion was developed, and its formation was analyzed quantitatively over time. Thirty-nine Wistar rats were randomly divided into intact control (n = 3) and experimental (n = 36) groups. The latter was equally divided into three groups according to the experimental intervention: fixed with deep bending of the knee joint (group I), fixed after incision of the capsule (group II), and fixed after exposure of the patellofemoral joint to artificial patellar subluxation (group III). All rats were subdivided according to their joint immobilization period (1, 2, or 4 weeks). Thereafter, the limited range of motion of the knee joint with (limited knee range of motion) and without (limited knee joint intrinsic range of motion) skin and muscles were measured. The lengths of adhesions of the anterior knee joint and posterior capsules were evaluated histologically. The limited intrinsic range of motion of the knee joint was found to be increased in groups II and III compared to that in group I 4 weeks after immobilization. Adhesions were confirmed within 1 week after immobilization in groups II and III. The length of the adhesions in group III was significantly longer than in other groups at 2 weeks and remained longer than in group I at 4 weeks. This model may contribute to the assessment of the adhesion process and development of new therapeutic avenues following trauma or surgical invasion.

## Introduction

Posttraumatic joint contracture (PTJC), an invasion near the joint, is an important target of postoperative management, as it secondarily limits patient mobility and reduces their quality of life [1]. The pathogenesis and changes over time must be understood in order to develop preventive and therapeutic methods for PTJC. Although involvement of the joint capsule has been reported as the main etiology of PTJC [2, 3], this has not been clarified. PTJC may share some of the same pathogenesis as immovable joint contracture, which is caused by the immobilization of a joint without trauma. However, this is clearly different, as it is associated with direct tissue damage and an accompanying inflammatory response. In general, PTJC induces more severe changes earlier than immovable-joint contracture. The promotion of tissue fibrosis and formation of adhesions between tissues are speculated to be the factors that lead to this outcome.

Postoperative adhesions occur frequently, and their associated complications are problematic in obstetrics, gynecology, and gastrointestinal surgeries. Several factors related to adhesion formation have been identified in adhesion research, such as bleeding and inflammation caused by incision and tissue desiccation, which induce tissue oxidation, collagen denaturation, and fibrosis [4–9]. However, in orthopedics, the formation and mechanism of tissue adhesion in PTJC remain unclear, warranting further research.

To elucidate the pathogenesis of PTJC, experimental animal models that reflect the clinical symptoms must be established. Animal models of PTJC have been developed using the knee and elbow joints of rabbits and rats [10–14]. However, in previous studies, the presence or absence of adhesions was assessed only subjectively [15]. In clinical practice, adhesions are encountered in this region, including in the suprapatellar bursa and patellofemoral joint [16]. A previous study reported that the immobilization of the knee joint in the flexed position stabilized clinically encountered joint adhesions [17]. However, no studies have quantitatively analyzed these lesions over time. Therefore, we evaluated the adhesion formation in the anterior knee joint using an improved animal model of PTJC tissue adhesion. This study aimed to quantitatively analyze the process of adhesion formation over time by modifying a surgical rat knee joint contracture model.

## Materials and methods

### Experimental design

Thirty-nine male Wistar rats (weight, 264 ± 36 g; age, 12 weeks) were randomly divided into an intact control group (n = 3) and an experimental group that underwent surgery (n = 36). Rats in the experimental group were divided equally into three surgical immobilization models (groups I, II, and III), as described below. Thereafter, the rats were randomly allocated to groups based on an evaluation period of 1, 2, or 4 weeks after the surgery (Table 1). Rats were housed in plastic cages in a controlled environment, allowing them to move freely and access food. No rats dropped out during the study period. This study was approved by the Animal Research Committee of Kyoto University (approval number: MedKyo19028), and all efforts were made to minimize suffering.

**Table 1:** Allocation of rats into groups.

Evaluation period	Group	Number of rats
1 week	Intact control	n = 1
	Group I	n = 4
	Group II	n = 4
	Group III	n = 4
2 weeks	Intact control	n = 1
	Group I	n = 4
	Group II	n = 4
	Group III	n = 4
4 weeks	Intact control	n = 1
	Group I	n = 4
	Group II	n = 4
	Group III	n = 4

### Surgical procedure

**Fig 1:**
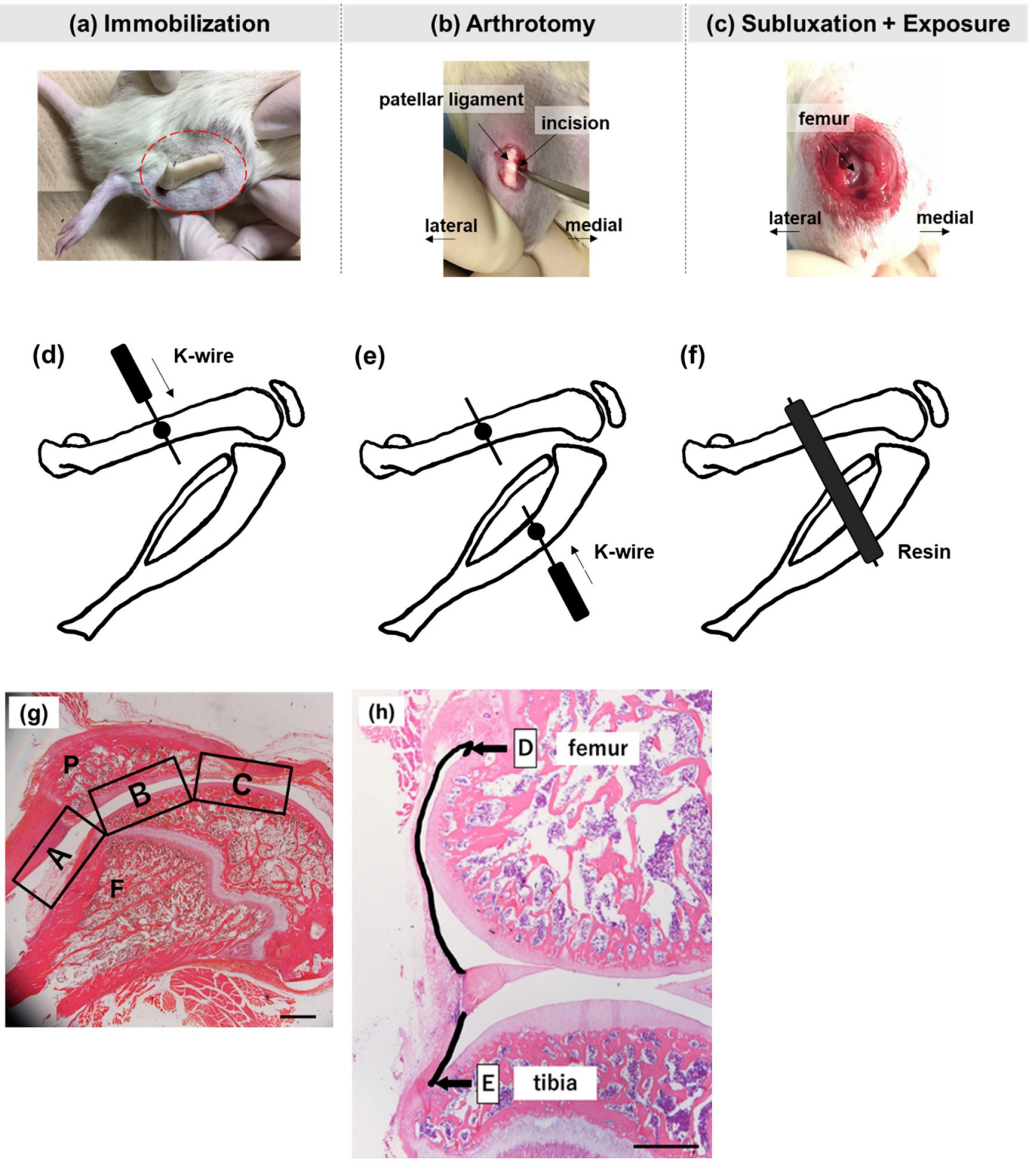
Surgical procedures. **(a)** Rat knee joint fixed with deep bending using Kirschner wire and resin; **(b)** fixed after incision of the inner joint capsule; **(c)** fixed after dislocation of the patella from the femoral pulley groove for 5 min. **A line drawing of the surgical fixation method. (d)** Insertion of K-wire into the femur **(e)** Insertion of K-wire into the tibia **(f)** Fixation using resin. (**g). Assessment regions of the adhesion length of the anterior knee joint in the histological image of a standard sagittal section with hematoxylin and eosin staining**. (A) The suprapatellar bursa. (B) The patellofemoral joint. (C) The distal part of the patella. P, patella; F, femur. Scale bar: 1000 μm. (**h). Microphotograph of the sagittal section of the medial region of the rat knee joint**. (D) Posterosuperior capsule length. (E) Posteroinferior capsule length. Hematoxylin and eosin staining. Scale bar: 1,000 μm.

Three surgical procedures were performed under 2% isoflurane inhalation anesthesia (Fig 1a–1c). The rats were transferred to their cages after surgery, and abnormal signs were monitored during the intervention period. All rats completed the experiment, and none died or experienced serious postoperative complications.

#### (a) Group I

Kirschner wires (K-wires) with a 0.8 mm (1-210-150-21, Iso Medical Systems, Tokyo, Japan) were inserted in the middle of the right femur and tibia of the rats after placing them in the abdominal position. The right knee was then fixed using resin in a deep flexural posture of approximately 140° ± 5° as previously reported [13] (Fig 1d–1f).

#### (b) Group II

After K-wire insertion, the medial joint capsule was unfolded (1-cm-long) using a surgical blade along the medial margin of the right patellar ligament, and the joint capsule and skin were closed using a 4–0 nylon suture (S15G04N-45, Bear Medic Inc., Tokyo, Japan). The right knee joints were subsequently fixed, as described for group I.

#### (c) Group III

After K-wire insertion, the medial joint capsule was unfolded as in group II. Thereafter, the patella was manually dislocated laterally from the femoral pulley groove for 5 min, and the joint capsule and skin were closed. The ligaments surrounding the joints were not severed. The right knee joints were fixed as described for group I.

Bleeding was controlled to the same degree as much as possible in each group and was eliminated using sterilized gauze during the operation. The fixed terms for each group were assumed to be 1, 2, and 4 weeks.

### Joint angle measurement

Angle analysis was performed on all the animals under 2% isoflurane anesthesia. After the K-wire was removed, the limited range of motion (ROM) of the right knee joint was measured with the skin and muscles intact (limited knee ROM). After sacrificing the animals, the limited knee joint ROM was measured without the skin and muscle (limited knee joint intrinsic ROM). The measurements were performed with the hip joint at approximately 90° flexion in the neutral spine position while pulling a leg at 0.49 N using a tension gauge (DS2 series, Imada, Toyohashi, Japan) according to the methods described in a previous study [13]. Furthermore, the angle between the femur and tibia was measured using a protractor at 5-degree increments. The knee extension limitation angle in this study was defined as the difference between 180° and the maximum extension angle.

### Histological analysis

#### Tissue preparation

The right knees were dissected following measurement of the joint intrinsic ROM. The knee joints were fixed in 4% paraformaldehyde overnight at an angle that appeared when the tension was removed from the extended position and decalcified in 10% ethylenediaminetetraacetic acid. The specimens were then embedded in paraffin. Sagittal sections 6 μm thick were prepared at sites every 200 μm from the center of the knee joint to the medial site using a microtome (RM2125 RTS, Leica, Nussloch, Germany). Sections were stained with hematoxylin and eosin (H&E) according to standard procedures. Images from each sample were quantified using the ImageJ software (National Institutes of Health, Bethesda, MD, USA). The remaining knee sections were stained with picrosirius red (Picrosirius Red Stain Kit, Polyscience Inc., Warrington, PA, USA) to visualize the collagen fibers in the adhesive area, according to the manufacturer’s instructions. Sections were observed under a polarized light microscope (Eclipse 80i, Nikon, Tokyo, Japan).

#### Adhesion length

Adhesion was defined as a pathological band of tissue formed ectopically across two surfaces inside the knee joint, and the continuity of the tissue was identified using H&E staining. The continuity of the tissue, which was not connected under normal conditions, was confirmed using magnified H&E-stained images. The accuracy was verified using scanning electron microscopy (SEM) results. To quantitatively evaluate adhesions, the anterior knee joint was divided into three parts (suprapatellar bursa, patellofemoral joint, and distal patella) in the sagittal section. The length of adhesions was evaluated at all medial condyle sites at 200-μm intervals. The mean adhesion length was calculated for three consecutive sections where the total adhesion length reached its maximum value (Fig 1g).

#### Capsule length

Posterior capsule length was measured as previously reported [17]. The superior and inferior posterior capsule lengths were measured separately (Fig 1h). The length of the posterior capsule was defined as the sum of these lengths.

#### SEM

Knee sections were deparaffinized in xylene and hydrated using a decreasing ethanol gradient. After pre-fixation with 2% glutaraldehyde at 4 °C for 2 h, tissues were postfixed in 1% osmium tetroxide for 2 h. The samples were dehydrated, dried, mounted on an aluminum stub, and coated using a JEC-3000FC ion sputter coater (JEOL, Tokyo, Japan). The specimens were observed under a JEOL JSM-7900F scanning electron microscope (JEOL, Tokyo, Japan).

### Statistical analysis

The JMP 15 Pro software (SAS Institute, Cary, NC, USA) was used for statistical analyses. Two-way analysis of variance (ANOVA) and post hoc Tukey’s Honestly Significant Difference test were performed to compare the three model groups in each evaluation period and the changes over time in each group. Correlation coefficients between the histomorphometric data and ROM were calculated using Spearman’s rank correlation analysis. All data are expressed as mean ± standard error. Statistical significance was set at *p* <0.05.

## Results

### Measurement of the joint angles

**Fig 2:**
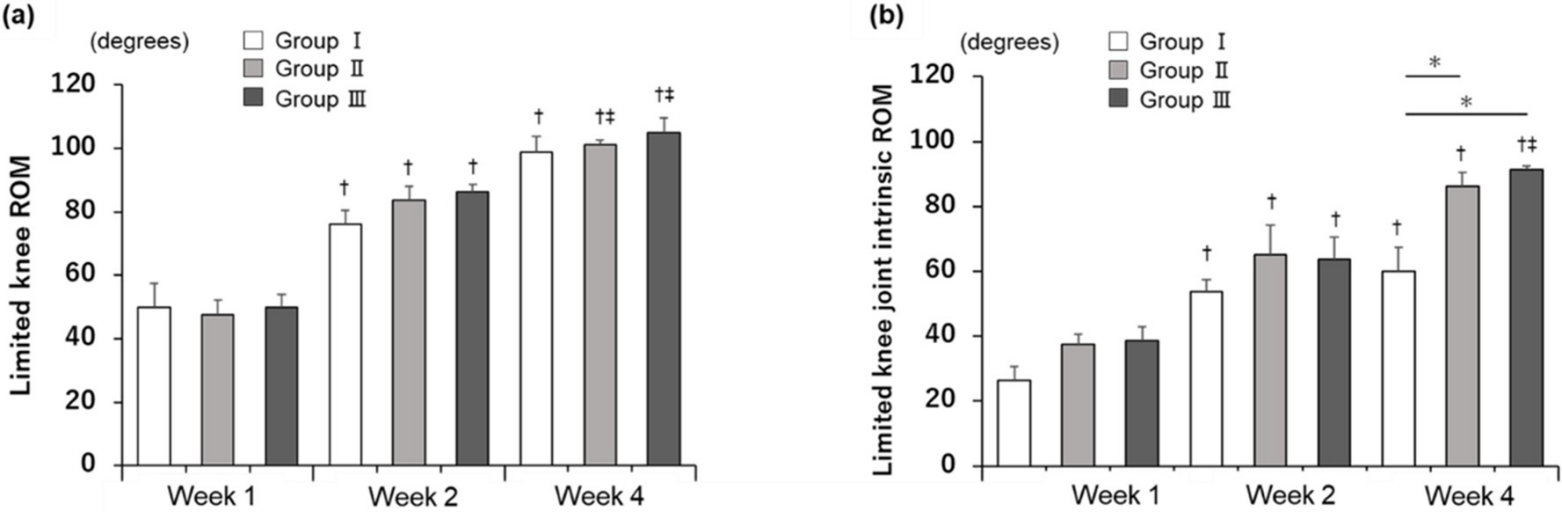
Knee joint angle at the point in each evaluation period. **(a)** Restriction of extension range of motion (ROM) involving the skin and muscles. A two-way ANOVA revealed significant effects by group (F[4, 31]: 56.18; *p* <0.001). **(b)** Restriction of extension ROM excluding the skin and muscles. Two-way ANOVA revealed significant effects by group (F[4, 31]: 26.67; *p* <0.0001). Data are expressed as mean ± standard error. n = 4 in each group. †*p* <0.05 (vs. 1 week), ‡*p* < 0.05 (vs. 2 weeks), *p <0.05 (vs. group I), §*p* <0.05 (vs. group II).

In the intact control group, the limited knee ROM and limited knee joint intrinsic ROM were 35° and 30°, respectively (S1 Table). Limited ROM increased significantly over time in all groups, except from 2 to 4 weeks in group I, with no significant differences among the three groups (*p* <0.05; Fig 2a). In addition, limited knee joint intrinsic ROM increased significantly over time in all groups except from 2 to 4 weeks in groups I and II, and was significantly higher in group II (86.3° ± 4.3°) and III (91.3° ± 1.3°) than in group I (60° ± 7.4°) at 4 weeks after immobilization (*p* <0.05; Fig 2b, S1 Table).

### Histological analysis

#### Adhesion observation

**Fig 3:**
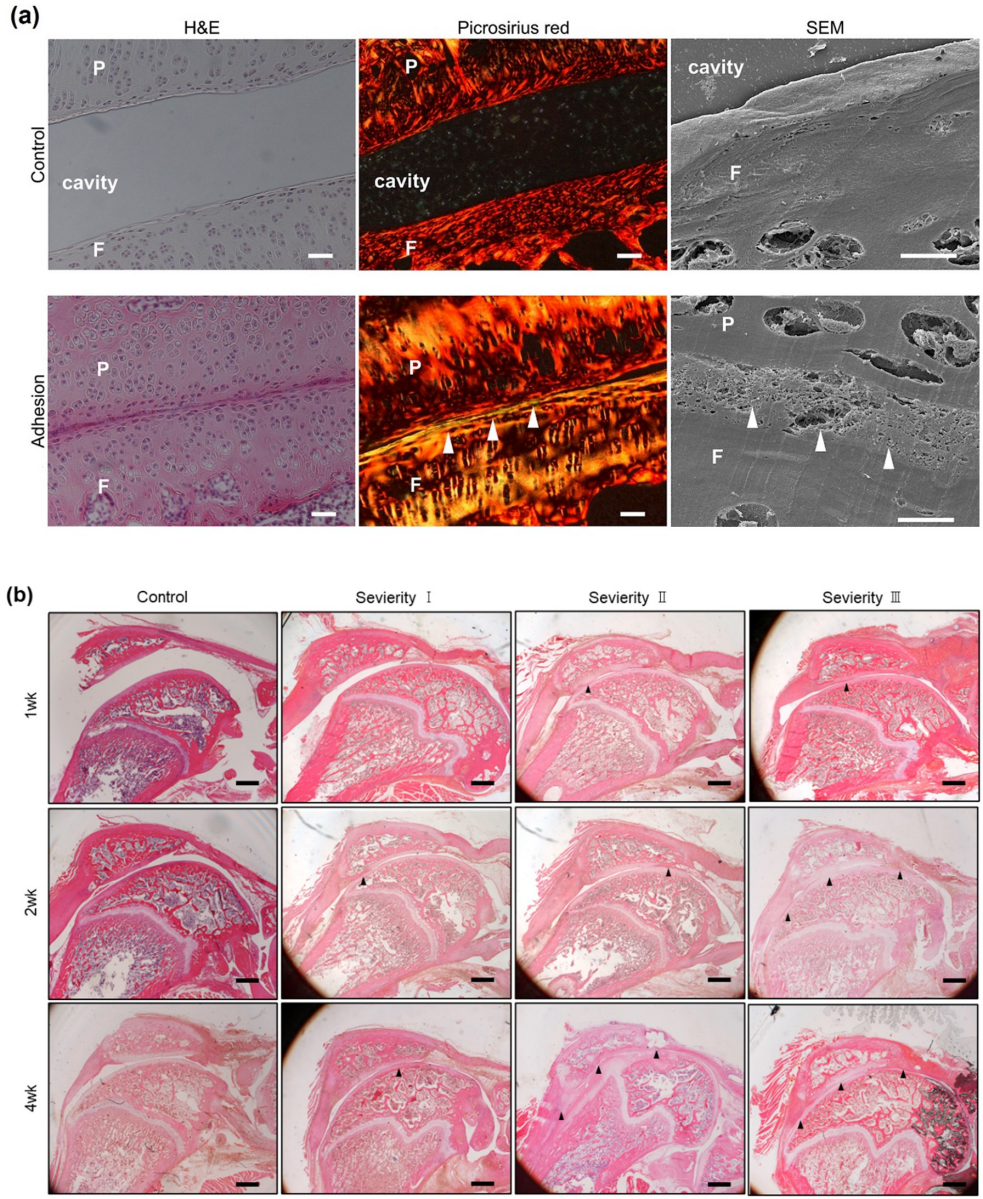
**a. Adhesion sites of the anterior knee joint. Hematoxylin and eosin (H&E), picrosirius red staining, and scanning electron microscopy (SEM) images of adhesion sites**. Upper panels: In the control group (13-week-old), the images show a well-organized structure with an articular cavity. Lower panels: In the experimental groups (picrosirius red staining: 4 weeks group III, SEM: 1 week group III), the images show disorganized dense collagen fiber structure (arrowheads) between the patella and femur. Scale bar: H&E staining, 100 μm; picrosirius red staining, 100 μm; SEM, 10 μm. **b. Changes in the histologic appearance of the adhesion of the knee joint with H&E staining**. The arrowheads show adhesion sites. P, patella; F, femur. Scale bar: 1,000 μm.

An articular cavity was not found at the adhesion sites, as confirmed by the magnified images of H&E staining, and the boundaries between the hyaline cartilage of the patella and femur and between the fibrous and cartilage tissue were unclear. Picrosirius red staining and SEM revealed the accumulation of dense and irregular collagen fibers at the adhesive sites (Fig 3a).

H&E staining revealed varying degrees of morphological adhesion in all the groups (Fig 3b). Adhesions were detected between the two synovium layers in the suprapatellar bursa and between the synovium and cartilage of the femur at a site distal to the patella. In addition, adhesions in the suprapatellar bursa and patellofemoral joint were observed 1 week after immobilization in groups II and III, but not in group I.

#### Adhesion length

**Fig 4:**
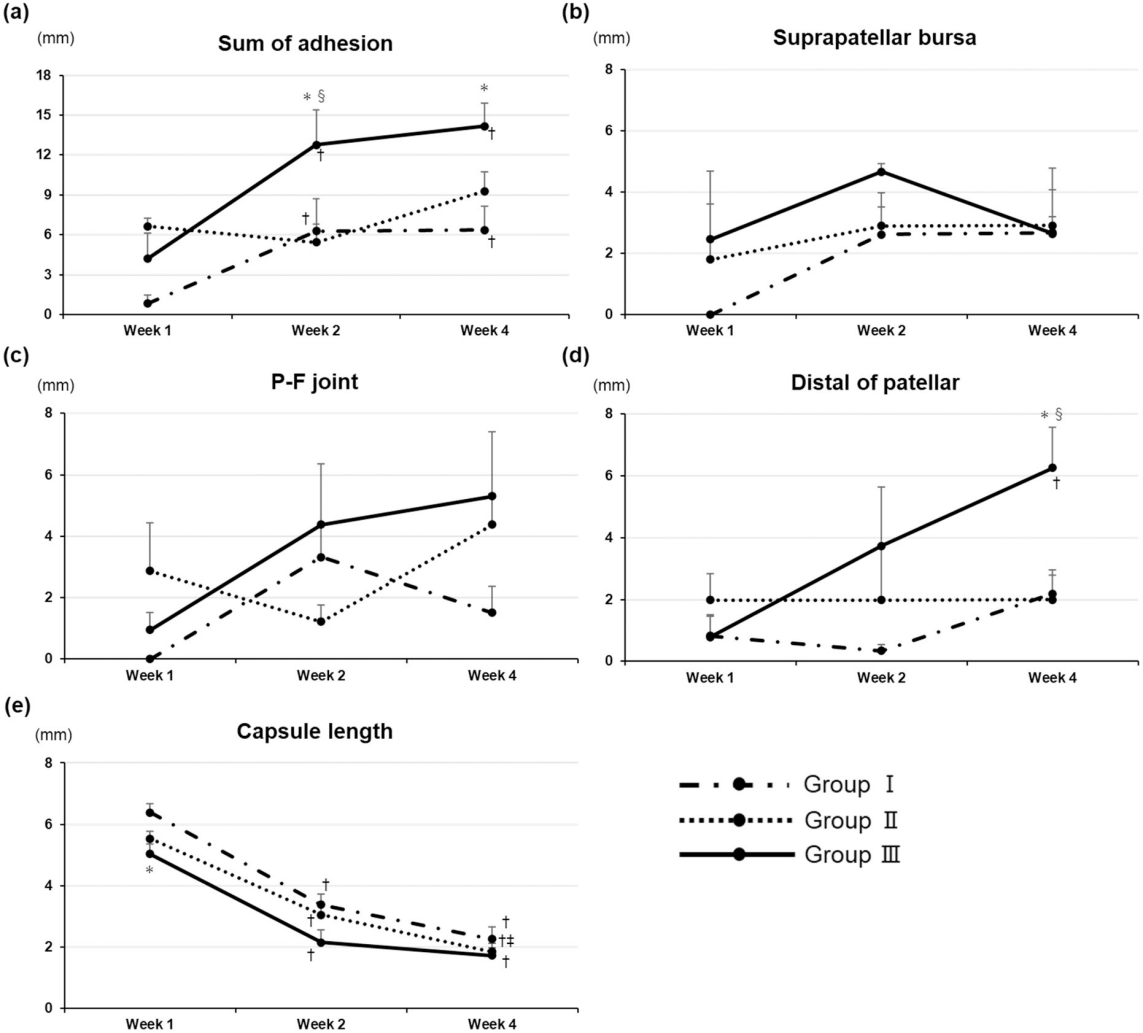
Length of knee adhesions and posterior capsule. **(a)** Adhesion length. Two-way ANOVA revealed significant intergroup effects (F[4, 31]: 7.71; *p* = 0.0002). **(b)** Suprapatellar bursa adhesion length. Two-way ANOVA revealed no significant intergroup effects (F[4, 31]: 1.99; *p* = 0.12). **(c)** Patellofemoral joint adhesion length. Two-way ANOVA revealed no significant intergroup effect (F[4, 31]: 2.24; *p* = 0.09). **(d)** Distal length of patellar adhesion. Two-way ANOVA revealed significant intergroup effects (F[4, 31]: 4.31; *p* = 0.0069). Data are expressed as mean ± standard error (n = 4 in each group). †*p* <0.05 (vs. 1 week), ‡*p* < 0.05 (vs. 2 weeks), *p <0.05 (vs. group I), §*p* <0.05 (vs. group II). **(e). Posterior capsule length after immobilization**. Two-way ANOVA revealed significant intergroup effects (F[4, 31]: 61.66; *p* < 0.0001). Data are expressed as mean ± standard error (n = 4 in each group). †*p* <0.05 (vs. 1 week), ‡*p* < 0.05 (vs. 2 weeks), *p <0.05 (vs. group I), §*p* <0.05 (vs. group II).

The total adhesion length significantly increased over time in groups I and III, except from 2 to 4 weeks (*p* <0.05; Fig 4a). The length of adhesion was significantly longer in group III than in the other groups at 2 weeks after immobilization (*p* < 0.05; Fig 4a). The length of adhesions in group III significantly increased 4 weeks after immobilization compared with that in group I (*p* <0.05; Fig 4a). The length of the sum of adhesions was significantly correlated with each ROM (length of the sum of adhesions vs. limited ROM, R: 0.54, *p* <0.001; vs. limited knee joint intrinsic ROM, R: 0.63, *p* <0.001). In the suprapatellar bursa and patellofemoral joint, no adhesions were found in group I 1 week after immobilization. In addition, no significant differences or changes over time were observed between the groups (Fig 4b, 4c). The length at the site distal to the patella was significantly longer in group III from 1 to 4 weeks and significantly longer in group III than in the other groups at 4 weeks after immobilization (*p* <0.05; Fig 4d).

#### Capsule length

The posterior capsule length significantly shortened over time in all groups, except from 2 to 4 weeks in groups I and III (Fig 4e). A week after immobilization, the posterior capsule length in group III was significantly shorter than that in group I (Fig 4e). However, no significant differences were detected between the groups at 2 or 4 weeks after immobilization (Fig 4e). The length of the posterior capsule was significantly correlated with each ROM (length of the posterior capsule vs. limited ROM, R = –0.89, *p* <0.001; vs. limited knee joint intrinsic ROM, R = –0.87, *p* <0.001).

## Discussion

The formation of adhesions between tissues is a likely cause of PTJC. Nevertheless, most studies on PTJC have focused only on soft tissues, including the capsule. Furthermore, there are no reports on the quantitative analysis of adhesion sites over time. In this study, we developed a rat model of knee joint adhesions, including medial parapatellar arthrotomy and exposure to lateral patellar dislocation, in addition to the conventional immobilization model [13] and quantitatively analyzed the process of adhesion formation over time. Clinically relevant adhesion formation was observed in the 1-, 2-, and 4-week immobilization groups. In addition, the knee joints of rats in group III were exposed to patellar subluxation for 5 min. The knee joint tissues in this group may have been desiccated, and the extensor apparatus may have caused minor trauma via patellar subluxation. To the best of our knowledge, no previous study has investigated the relationship between desiccation time and adhesion formation. Our results revealed that even a brief tissue exposure to subluxation promoted adhesion in the knee joint. This finding suggests that further investigation is necessary to examine the correlation between desiccation time and adhesion formation.

Knee extension restrictions increase with immobilization duration [18]. Our results are consistent with these findings and support the validity of the fixation method used in this study. The factors of joint contracture pathology are mainly divided into myogenic and joint intrinsic factors [3], including the contribution of decreased myogenic contracture and increased joint intrinsic contracture as the duration of immobilization increases [13]. In this study, limited ROM tended to increase in all groups; however, there were no significant differences between the groups. We considered that myogenic limitation affected ROM because the joint angles increased when the myogenic limitation was removed. Myogenic limitation predominates during the first 4 weeks of immobility [12]. The change that occurred when the intrinsic joint limitation was removed with an unremoved myogenic limitation was unidentified (immeasurable). However, the present results show that myogenic limitation did not change when intrinsic joint limitation increased with adhesion formation. Therefore, we believe that myogenic limitations affect the ROM. In addition, we consider that the myogenic limitation of the limited ROM was only minimally affected because the operative wound was the anterior knee, and there was little damage to the knee flexor muscles. Limitation of motion is a primary response to the formation of adhesions and proliferation of capsular connective tissue [19]. The contribution of shortening the posterior capsule length to ROM was high, and adhesion formation affected the ROM to a certain extent. The synergistic effects of soft tissue fibrosis, such as capsule and adhesion formation, may limit joint mobility. Importantly, intraarticular adhesions combined with arthrotomy and joint desiccation were observed within 1 week after immobilization, suggesting that preventive therapeutics should be administered as early as possible.

Picrosirius red staining and SEM revealed that the boundaries between adjacent tissues were unclear owing to the accumulation of dense and irregular collagen fibers. The rats in group II experienced direct tissue damage, which led to increased adhesion formation. Furthermore, joint desiccation significantly increased adhesion in group III rats compared with group II rats. The length of the adherent area did not show consistent changes over time, particularly in group II. We considered that the inconsistent results occurred because the individual differences in adhesion formation were relatively large, and the sample size was small. However, the tendency of change in each group was observed by the sum of the adhesions that merged each part, and we believe that this could reflect the characteristics of the models. A previous study using a rat knee immobilization model revealed that knee joint flexion contractures could restrict full joint extension. These were also caused by fibrosis of the adjacent soft tissues, including the synovial membrane and fibrous proliferation of the infrapatellar fat pad [3]. Another study reported the fibrosis and atrophy of adipose cells after joint immobilization [20]. Synovial inflammation caused by immobilization in rat knees revealed marked inflammatory changes, followed by mononuclear cell infiltration and fibrosis [21]. Additionally, tissue invasion, bleeding, and drying can result in adhesion formation [22]. The mechanism of adhesion formation in long-term open wounds during surgery is related to the subsequent fibrin deposition [13–15]. Another study revealed that mesothelioma cells produce transforming growth factor-β via interleukin 6 after fibrin deposition and that mesothelioma cells become the main body of adhesive formation by transforming growth factor-β [9]. As mentioned previously, because significant adhesions formed distal to the patella in this study, fibrin deposition through cytokines produced by adipocytes or synovial inflammation might easily occur after arthrotomy and joint desiccation, thus becoming an important factor for adhesion formation in the knee joint.

This study had several limitations. First, the effects of remobilization were not investigated. Examining stable adhesion, which can contribute to the observed stiffness, remains challenging. A previous study reported that 4 weeks of joint immobilization caused capsule adhesion and a restriction of joint motion which was irreversible even after subsequent remobilization for 16 weeks [23]. This indicates that although smaller and clinically unimportant adhesions may lead to recovery through natural processes, knee joint immobilization causes irreversible changes in adhesions. Second, although we defined an adhesion as a pathological band of tissue formed ectopically across two surfaces inside the knee joint, the toughness of the adhesion was not mechanically investigated. Thus, the tightness of the adhesions is unclear. Third, intraarticular adhesions were investigated using a small sample size. Therefore, caution should be exercised when generalizing the results. Fourth, the knees of rodents are not fully extended, and thus differ from those of humans. In addition, humans are bipedal, whereas rodents are quadrupedal. These differences require careful interpretation of the results and future applications in clinical practice. Finally, this study focused on the morphological analyses of knee joint adhesions to establish grounds for the development of new models; however, detailed molecular mechanisms, including the role of inflammatory cytokines and changes in collagen type, were not assessed. The molecular mechanisms over time and a series of inflammatory- and fibrosis-related adhesion markers should be investigated in future studies.

In conclusion, we developed a rat model of knee joint adhesion. To the best of our knowledge, this is the first study to develop an appropriate animal model of PTJC tissue adhesion in the knee joint and to quantitatively analyze the adhesion sites over time. These animal models can be used for various purposes at different periods. Furthermore, these models may help to verify the adhesion process or develop new therapeutic avenues, such as rehabilitation following trauma or surgical invasion of the musculoskeletal system.

## Supporting information

S1 Table Raw data of a limited range of motion of maximum knee extension after immobilization. (DOCX)
