# Supplementary material for: Development of a novel model for intraarticular adhesion in rat knee joint
Source: PLoS One. 2023 Sep 21;18(9):e0292000. doi: 10.1371/journal.pone.0292000 (PMC10513256; doi:10.1371/journal.pone.0292000)
Supplement: S1 Table — (DOCX) [file pone.0292000.s001.docx]

**S1 Table. Raw data of a limited range of motion (ROM) of maximum knee extension after immobilization**

| Sample number | Evaluation period | Group | Limited knee ROM (degrees) | Limited knee joint intrinsic ROM (degrees) |
| --- | --- | --- | --- | --- |
| 1 | 1 week | Intact control | 35 | 30 |
| 2 | 1 week | I | 35 | 25 |
| 3 | 1 week | I | 45 | 30 |
| 4 | 1 week | I | 70 | 15 |
| 5 | 1 week | I | 50 | 35 |
| 6 | 1 week | II | 40 | 35 |
| 7 | 1 week | II | 40 | 40 |
| 8 | 1 week | II | 60 | 45 |
| 9 | 1 week | II | 50 | 30 |
| 10 | 1 week | III | 60 | 50 |
| 11 | 1 week | III | 40 | 35 |
| 12 | 1 week | III | 50 | 30 |
| 13 | 1 week | III | 50 | 40 |
| 14 | 2 weeks | Intact control | 35 | 30 |
| 15 | 2 weeks | I | 80 | 60 |
| 16 | 2 weeks | I | 75 | 50 |
| 17 | 2 weeks | I | 85 | 45 |
| 18 | 2 weeks | I | 65 | 60 |
| 19 | 2 weeks | II | 95 | 90 |
| 20 | 2 weeks | II | 85 | 65 |
| 21 | 2 weeks | II | 80 | 60 |
| 22 | 2 weeks | II | 75 | 45 |
| 23 | 2 weeks | III | 85 | 80 |
| 24 | 2 weeks | III | 90 | 70 |
| 25 | 2 weeks | III | 90 | 55 |
| 26 | 2 weeks | III | 80 | 50 |
| 27 | 4 weeks | Intact control | 35 | 30 |
| 28 | 4 weeks | I | 90 | 40 |
| 29 | 4 weeks | I | 110 | 75 |
| 30 | 4 weeks | I | 90 | 60 |
| 31 | 4 weeks | I | 105 | 65 |
| 32 | 4 weeks | II | 100 | 90 |
| 33 | 4 weeks | II | 100 | 75 |
| 34 | 4 weeks | II | 100 | 95 |
| 35 | 4 weeks | II | 105 | 85 |
| 36 | 4 weeks | III | 100 | 90 |
| 37 | 4 weeks | III | 95 | 90 |
| 38 | 4 weeks | III | 110 | 95 |
| 39 | 4 weeks | III | 115 | 90 |
